# Frequent Occurrence of Simultaneous Infection with Multiple Rotaviruses in Swiss Pigs

**DOI:** 10.3390/v14051117

**Published:** 2022-05-23

**Authors:** Sibylle Baumann, Titus Sydler, Giuliana Rosato, Monika Hilbe, Dolf Kümmerlen, Xaver Sidler, Claudia Bachofen

**Affiliations:** 1 Institute of Virology, Vetsuisse Faculty, University of Zurich, 8057 Zurich, Switzerland; sibylle.baumann@icloud.com; 2 Institute of Veterinary Pathology, Vetsuisse Faculty, University of Zurich, 8057 Zurich, Switzerland; tsyd@vetpath.uzh.ch (T.S.); g.rosato@access.uzh.ch (G.R.); hilbe@vetpath.uzh.ch (M.H.); 3 Division of Swine Medicine, Department of Farm Animals, Vetsuisse Faculty, University of Zurich, 8057 Zurich, Switzerland; dkuemmerlen@vetclinics.uzh.ch (D.K.); xsidler@vetclinics.uzh.ch (X.S.)

**Keywords:** porcine rotavirus, multiple infections, genotypes, Switzerland

## Abstract

Rotavirus (RV) infections are the most important viral cause of diarrhea in piglets in Switzerland and are thought to cause substantial economic losses to the pig industry. However, no data are available on the occurrence and dynamics of the main porcine RV species, namely RVA, RVB, and RVC, and the diversity of the circulating strains. We therefore tested fecal samples from a cross-sectional (*n* = 95) and a longitudinal (*n* = 48) study for RVA, RVB, and RVC by real-time RT-PCR and compared the results of the cross-sectional study to postmortem findings. In addition, eight samples were fully genotyped by using next-generation sequencing. In the cross-sectional study, triple RV infections significantly correlated with diarrhea and wasting and were most frequent in the weaned age group. In the longitudinal study, the shedding of RV peaked one week after weaning and decreased thereafter. Here, mainly double infections were seen, and only a few animals showed diarrhea. The full-genome sequencing revealed a genotype pattern similar to other European countries and, importantly, co-infection by up to four RVA strains. Our results imply that the weaning of piglets may trigger not only RV shedding but facilitate co-infection of multiple RV species and strains in the same host.

## 1. Introduction

The genus *Rotavirus* is classified as a member of the *Reoviridae* family, and its genome consists of 11 segments of double-stranded RNA enclosed in a triple-layered virus particle. The genome encodes six structural proteins (VP1–4, VP6, and VP7) and five or six nonstructural proteins (NSP1–6). RVs are one of the most common pathogens associated with acute gastroenteritis in infants, both in humans and animals worldwide [1,2]. Especially in suckling and weaned pigs, RV infections can cause villus atrophy in the small intestine, leading to malabsorption and diarrhea, causing economic losses related to growth impairment and increased mortality [2,3,4,5]. The susceptibility to clinical disease decreases as the age progresses [5,6]. Nevertheless, diarrhea is a multi-factorial and multi-etiological disease and mainly the result of a combination of poor maternal antibody protection and a high infection pressure of enteric pathogens [5,7]. Based on the antigenic characteristics of VP6 [8], RVs are currently classified into nine species, A to J (https://talk.ictvonline.org/taxonomy/; visited on 8 April 2022). However, two more tentative species (RVK and L) have recently been described in shrews from Germany [9,10]. In pigs, the species A, B, C, and H have been described, as well as the proposed species E [2,11,12,13]. *Rotavirus A* (RVA), followed by *Rotavirus C* (RVC) and *B* (RVB), are the most frequent species [2,12].

RV occurs either alone or in mixed infections of multiple RV species. Such simultaneous infections with multiple RV species are described frequently in pigs [14,15,16]. Mixed infections were particularly often observed in weaned pigs [15,17]. Co-infection of multiple RV species was associated with diarrhea and may intensify the severity of the clinical signs [14,18]. However, mixed infections have also been detected in piglets without clinical signs [15]. In addition to inter-species co-infections, also intra-species co-infections, e.g., of several RVA strains concurrently, have been described [13,19]. The clinical impact of these multiple infections is not known. However, it is known that, due to the segmented genome of RV, such mixed infections offer abundant opportunity for reassortments and may give rise to strains with novel characteristics [19,20]. RVA, RVB, RVC, and RVH have not only been detected in pigs, but also in humans; thus, zoonotic interspecies transmissions are possible and may give rise to emerging RVA genotypes, such as the G9 strains that are globally emerging in humans and are thought to result from pig-to-human spillover [21]. While co-infections with multiple RVA strains are important, not much is known about how frequent and under what circumstances they occur. The detection is hampered by the fact that genotyping is usually based on the Sanger sequencing of PCR products resulting in only one dominant sequence or in mixed sequences with nucleotide ambiguities. Next-generation sequencing (NGS) may overcome this problem, either by targeted resequencing of PCR products [19] or by direct metagenomic sequencing of the sample material [13].

RV strains have traditionally been classified using only the two outer capsid proteins VP7 and VP4, defining the G (glycoprotein) and P (protease sensitive) genotypes, respectively [6]. To date, at least 41 G types and 57 P types have been described for RVA (https://rega.kuleuven.be/cev/viralmetagenomics/virus-classification/rcwg; accessed on 13 February 2022). However, in 2008, the extended full-genome sequence classification system for RVA strains was proposed which is based on the sequence data of all 11 gene segments [22,23]. To each segment (VP7-VP4-VP6-VP1-VP2-VP3-NSP1-NSP2-NSP3-NSP4-NSP5), a specific range of genotypes can be assigned, represented by letters (G-P-I-R-C-M-A-N-T-E-H) and numbers, e.g., A1–24 for NSP1. The range of circulating genotypes was shown to influence vaccine efficiency in humans, as heterotypic strains are less well neutralized than strains which are of the same P/G genotype as the vaccine strains [24]. Furthermore, full-genome genotyping may unravel animal origin of genome segments other than VP7 and VP4, thereby revealing previous interspecies transmissions [19,25,26].

Data on RV strains circulating in Switzerland are scarce. In Swiss children examined in two hospitals in 2010, G1–4, G9, and P [4,8] were found [25], but there are no data available on porcine RV strains. In Switzerland, the pig health status is high compared to that in other European countries. In the absence of important epizootic diseases, such as those caused by porcine reproductive respiratory syndrome virus (PRRSV) and transmissible gastroenteritis virus (TGEV), RV is considered the most important viral cause of diarrhea in Swiss piglets (https://www.blv.admin.ch/blv/de/home/tiere/tiergesundheit/frueherkennung/pathopig.html; accessed on 8 April 2022). However, nothing is known about the dynamics of RV species in Swiss pig herds and the genotypes present. The aim of this study was, therefore, to obtain first data on the occurrence of the RV species A, B, and C, the dynamics of mixed RV infections in different age groups and the genetic diversity within the RVA species circulating in Swiss pigs. The resulting data suggest that the diversity of porcine RV in Switzerland is high and that, in contrast to other porcine viruses, the range of genotypes is similar to the one in other European countries. Furthermore, RV mixed infections seem to be frequent and may be triggered by weaning.

## 2. Materials and Methods

### 2.1. Samples

#### 2.1.1. Cross-Sectional Study

Archived porcine fecal samples stored at −20 °C and formalin fixed intestinal samples from freshly euthanized pigs were provided by the Institute of Veterinary Pathology of the University of Zürich. The samples originated from pigs submitted for necropsies within the “PathoPig” project. The Swiss Federal Food Safety and Veterinary Office launched this project in 2014 to improve animal health by financially supporting postmortem analyses of pigs from herds with health problems [26]. Overall, individual samples of 95 pigs from 55 different farms, sampled between 2018 and 2020, were used for our study, including 31 suckling piglets, 37 weaned pigs, and 27 fattening pigs. The samples originated from 69 pigs with and 26 pigs without diarrhea. The inclusion criterium was the availability of intestinal samples that were formalin-fixed within max 1 h after euthanasia to allow the preservation of the intestinal morphology, especially of the superficial jejunal enterocytes.

#### 2.1.2. Longitudinal Study

Piglets from five different litters of the same farm were followed from weaning in week 4 until week 9 of life for an unpublished pilot study on zinc oxide uptake (a traditional feed additive only allowed in very limited concentrations in Switzerland) and retrospectively analyzed for RV. It was known that RV is circulating on this small breeding farm, but no increased problems with diarrhea were reported. The farm had no history of any specific intestinal pathogens after weaning. The first samples were taken just prior to the separation of the piglets from their mothers. At this timepoint, fecal samples were taken from each piglet and from the five sows. Pen floor samples were taken from the empty weaning pens and from the floor of the sow pens, as described previously [27]. Piglets were individually ear-tagged for identification during the study. The 48 piglets were then evenly distributed into two weaning pens, individual fecal samples were taken from the rectum once a week (except in week 7), and piglets were clinically examined for signs of diarrhea. After week 6, the pens were getting too small, and 20 animals were removed from the study. One animal had to be euthanized between week 5 and 6 and another one after week 8 for reasons unrelated to the study. The samples were stored at −20 °C until further processing.

### 2.2. Postmortem Analyses

Samples from the cross-sectional study were categorized by age of the pigs: suckling piglets (0.4–<5 weeks), weaned pigs (5–<10 weeks), and fattening pigs (10–36 weeks). In addition to age, postmortem findings that may be related to RV infection, such as diarrhea, wasting and shortening of the intestinal villi, were assessed. Diarrhea was rated macroscopically by the pathologist with yes or no. To determine the presence of wasting, the body weight was determined prior to necropsy and compared to the expected weight of the respective age group, as determined by the literature [28] and the experience of the pathologists. If the body weight was <2/3 of the expected weight, it was rated as wasting. Fixed intestinal segments were routinely processed, embedded in paraffin, sliced into 5 μm–thick sections, and stained with hematoxylin and eosin for light microscopy measurements of the villus height and crypt depth by pathologists of the Institute of Veterinary Pathology. Pigs younger than one week were expected to have a minimum villus–crypt ratio (VCR) of 7.5:1, pigs between one and four weeks were expected to have a minimum of 2.5:1, and pigs older than 10 weeks were expected to have a minimum of 2:1 (personal communication T.S. and [29]). If the VCR was below this ratio, villi-shortening was further graded as mild, moderate, or severe. An example for the histological measurement is provided in Supplementary Figure S1.

### 2.3. RNA Extraction

#### 2.3.1. Viral RNA Mini Kit

To gain RNA for subsequent real-time RT-PCR, fecal samples were defrosted and well mixed. A total of 100 mg of feces was weighed in a 2 mL Eppendorf tube, and 1000 µL PBS was added. Subsequently, the samples were homogenized in the TissueLyser II (Qiagen, Hilden, Germany) for 1 min with a frequency of 20 Hz and then centrifuged for 3 min at 16.000× *g*. The resulting supernatant was removed for RNA extraction by using the QIAamp Viral RNA Mini Kit (Qiagen, Hilden, Germany) according to the manufacturer’s manual but omitting the addition of carrier RNA. For the elution step, 50 µL of nuclease-free water was used. For each batch of RNA extraction, a negative extraction control was included, using water instead of feces. The RNA was stored at −20 °C until further processing.

#### 2.3.2. Phenol–Chloroform RNA Extraction

The phenol–chloroform method was used to prepare RNA for subsequent next-generation sequencing. Briefly, in a 2 mL tube, 1 mL of RNA extraction buffer (0.5% NP-40, 150 mM NaCl, 1.5 mM MgCl_2_, and 10 mM Tris-HCL, pH 7.4) was added to 400 mg of well-mixed defrosted fecal sample. The sample was homogenized for 30 s at 20 Hz in the TissueLyser II (Qiagen, Hilden, Germany) and, after centrifugation for 1 min at 16.000× *g,* 500 µL of the supernatant was transferred to a new 2 mL tube. Then 500 µL acid phenol–chloroform–IAA (125:24:1, pH 4.5) (Invitrogen™, Thermo Fisher Scientific, Waltham, MA, USA) was added, and the sample was vortexed for 20 s and centrifuged for 15 min at 16.000× *g*. The aqueous phase was carefully removed and transferred into a new 2 mL tube. Again, 500 µL acid phenol–chloroform–IAA was added, and the previous steps were repeated. After adding 50 µL of sodium acetate (3 M, pH 5.2, autoclaved), 3 µL of ultra-pure glycogen (20 µg/µL) (Invitrogen™, Thermo Fisher Scientific, Waltham, MA, USA), and 1 mL of 100% ethanol, the tube was vortexed, incubated at −80 °C for 30 min, and centrifuged for 30 min at 16.000× *g* at 4 °C to pellet the RNA. The pellet was washed once with 1 mL of 75% ethanol, dried in a laminar flow work bench, and resuspended in 60 µL of nuclease-free water. The RNA was stored at −20 °C until further processing.

### 2.4. Multiplex Real-Time RT-PCR Assays

All samples were screened for RVA, RVB, and RVC individually. For the detection of RVA, the commercial Swine Enteric Panel TGEV/PEDV/PRV-A was used together with the Path-ID™ Multiplex One-Step RT-PCR Kit^®^ (both Thermo Fisher Scientific, Waltham, MA, USA) according to the manufacturer’s manual. The kit allowed the concurrent recognition of RVA, porcine epidemic diarrhea virus (PEDV), and TGEV. For RVB and RVC detection, the Path-ID™ Multiplex One-Step RT-PCR Kit^®^ (Thermo Fisher Scientific, Waltham, MA, USA) was used with primers and probes described by Marthaler et al. [12]. Reactions were run on a QuantStudio™ 7 Flex Real-Time PCR System (Applied Biosystems™, Thermo Fisher Scientific, Waltham, MA, USA). The number of amplification cycles necessary to reach exponential DNA amplification (Ct value) was used to gain a semi-quantitative indication of the RNA level present at the beginning. In short, the lower the Ct value, the more RNA is present in the testing material.

### 2.5. Next-Generation Sequencing (NGS) and Genotyping

RVA positive samples from the cross-sectional study with a Ct value <33 and sufficient sample volume left were subjected to next-generation sequencing for full-genome determination. In addition, two pigpen-floor swab samples from a previous study on hepatitis E virus [27] were included, resulting in 29 samples.

After RNA extraction by using the phenol–chloroform method, reverse transcription, second strand synthesis, and amplification were performed by following an in-house protocol established by Kubacki et al. [30]. Afterward, the DNA concentration was measured by a Qubit^®^ fluorometer (Thermo Fisher Scientific, Waltham, MA, USA). Subsequently, samples were diluted with EB buffer to a final concentration of 3 ng in 58 µL, and the E220 Focused-ultrasonicator (Covaris, Woburn, MA, USA) was used to fragment the DNA to 500 base pairs (bp). Libraries were prepared with the NEBNext^®^ Ultra™ II DNA Library Prep Kit for Illumina^®^ (New England Biolabs, Ipswich, MA, USA) according to the manufacturer’s manual. The NovaSeq 6000 (Illumina, San Diego, CA, USA) was used for paired-end sequencing with a read length of 150 bp and at the Functional Genomics Center Zurich (FGCZ). Phi X Control v3 Library (Illumina, San Diego, CA, USA) was used as a quality control. The generated sequences were quality checked and analyzed in previously established de novo and reference guided assembly pipelines [31,32]. In short, quality checked reads were assembled by using metaspades (v3.12.0) and the de novo generated contigs compared to the NCBI non-redundant database using blastn (v2.8.1+) (https://blast.ncbi.nlm.nih.gov/Blast, accessed on 11 December 2021), annotated using the best blastn hits and mapped back to the assembled sequences using bwa (v0.7.17) mem (https://github.com/lh3/bwa, visited on 11 December 2021). De novo contigs of >500 nucleotides (nt) length were used for reference-based re-assembly using SeqMan NGen 17 software (Lasergene DNA star, Madison, WI, USA) with high stringency settings in order to determine the number of matching reads and visually control the alignments.

The online RVA genotyping tool RotaC [33] was used for genotyping (accessed on 11 December 2021 on https://www.viprbrc.org/). It follows the guidelines for full-genome genotyping of Rota A viruses recommended by the Rotavirus Classification Working Group (RCWG) [22]. All fully genotyped sequences were uploaded to GenBank (accession numbers OM982707–OM982822; details are provided in Supplementary Table S1), and the respective NGS raw data were submitted to SRA (BioSamples SAMN26634154– SAMN26634161).

### 2.6. Phylogenetic Analysis

For the phylogenetic analysis of the full-length and near-complete coding sequences (CDSs) of all segments with more than 95% coverage, the MEGA X software was used [34] to calculate the best-fitting model. A maximum likelihood tree with 500 bootstraps was constructed by using the General Time Reversible model with a discrete Gamma distribution with Invariant sites. For each sequence, full CDS reference genomes were downloaded from the NCBI rotavirus sequence database based on BLAST analysis, whereby the three most closely related sequences of porcine and human origin (reported as “host” by the database) of the respective genotype were included. In some cases, closely related sequences of other animal hosts were also included. In addition, all segments of three major porcine reference strains (OSU, Gottfried, YM), as well as of the two human isolates Wa and SD-1 that represent the two major RVA genogroups, were included. Of the AU-1 reference strain, representing the minor genogroup, only the VP4 and VP7 sequences were used. As representatives of European porcine RVA we added all segments of five well-characterized Belgian strains: VR277 from 1977 and 12R002, -005, -006, and -041 from 2012 [35].

### 2.7. Statistical Analysis

For the statistical analysis, the NCSS 10 statistical software was used (NCSS LLC, East Kaysville, UT, USA). The *p*-values below 0.05 were considered significant. To compare proportions of animals infected by increasing numbers of RV species with regard to necropsy findings or age groups, a contingency table was calculated, followed by pairwise comparisons by Chi-square testing.

## 3. Results

### 3.1. Cross-Sectional Study

**Figure 1:**
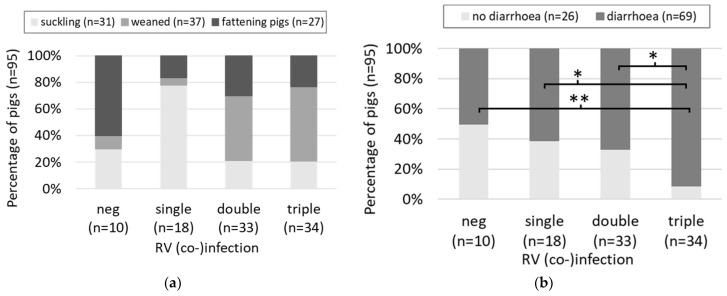

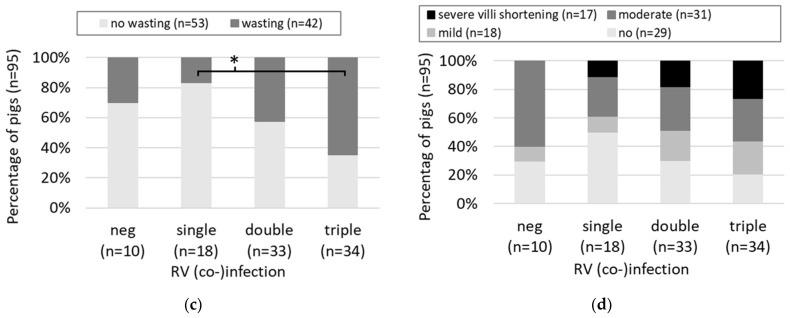
The percentage of animals from the cross-sectional study (*n* = 95) infected with none, a single, two, or three RV species and (**a**) grouped by age group, (**b**) by absence/presence of diarrhea, (**c**) by absence/presence of wasting, and (**d**) by degrees of VCR, are shown. Statistically significant differences are indicated by asterisks (* *p* < 0.05, and ** *p* < 0.005).

A total of 95 samples from 31 suckling piglets, 37 weaned pigs, and 27 fattening pigs were tested for RVA (including TGEV and PEDV), RVB, and RVC. The age range was between newborn and 36 weeks, with 50% of the animals being between 5 and 10 weeks of age. All animals were negative for TGEV and PEDV. Only 10 animals (11%) were negative for all RV, while 18 (19%) were infected with a single RV species. The majority of animals was positive for two (*n* = 33, 35%) or three (*n* = 34, 36%) RV species (Figure 1). RV negative animals belonged mainly to the age group of the fattening pigs (6 out of 10 animals), three animals were suckling pigs, and only a single weaned pig was negative for all RV species (Figure 1a). In contrast, the animals with double and triple RV infection were mainly weaned pigs, with 48% and 56%, respectively. Interestingly, single infections were primarily observed in suckling piglets (78%). However, due to some groups counting less than five animals, a reliable statistical analysis was impossible. Overall, 69 animals (73%) showed diarrhea. With the increasing number of (co-)infecting RV species, the percentage of animals with diarrhea increased from 50% in RV negative animals to 91% in triple-infected cases (Figure 1b). Triple-infected animals were significantly more frequently affected by diarrhea than negative, single-infected, and double-infected animals. The picture was similar for wasting. In 42 cases, the body weight was below 2/3 of the expected weight. However, here, only the difference between triple and single infections was statistically significant, with 65% of the triple-infected pigs but only 17% of the single-infected pigs showing signs of wasting (Figure 1c). As for the age groups, the statistical comparison of VCR degrees in negative, single-, double- and triple-infected animals was not possible due to group sizes below 5. However, severe villi shortening was not observed in RV-negative animals, and the number of animals with severe villi shortening increased stepwise with the number of co-infecting RV species, with the highest percentage (26%) in triple-infected animals (Figure 1d).

**Figure 2:**
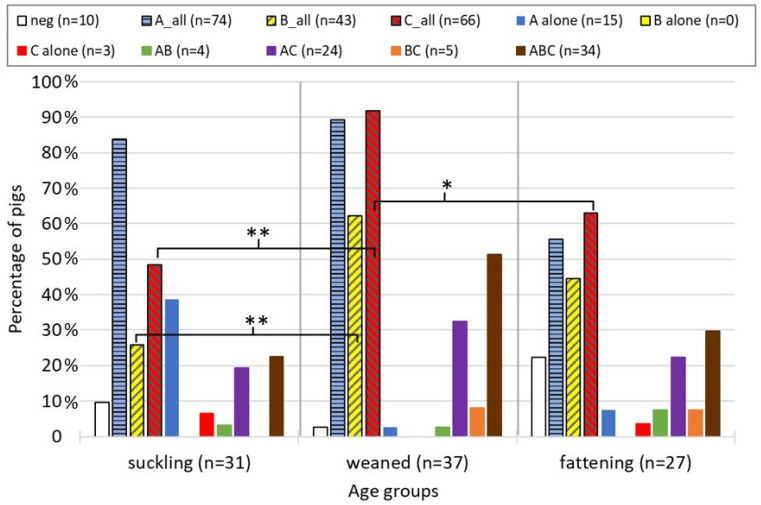
The percentage of pigs from the cross-sectional study (*n* = 95) (co-)infected with different types of RV species and grouped by age group is shown. The “_all” indicates that all samples positive for this RV species are included in this group (=overall result), independent of co-infecting species. Statistically significant differences are indicated by asterisks (* *p* < 0.05, and ** *p* < 0.005).

In 67 cases (71%), pigs showed coinfections of several RV species. Most frequently observed were triple infections of RVA, RVB, and RVC (36%) and double infections of RVA and C (25%). Single infections with RVB alone were not observed, and double infections of RVB and RVC, as well as single infections with RVC, were rare (5% and 3%, respectively). Interestingly, single infection with RVA was clearly dominant in suckling pigs (12 animals) but rare in weaned and fattening pigs (one and two animals, respectively) (Figure 2). In contrast, in weaned pigs, triple infections and RVA and RVC double infections were prevailing. While the group sizes were often too small for statistical comparison between the different types of (co-)infections, they were sufficiently large when using only the overall real-time RT-PCR results for RVA, RVB, and RVC, independent of co-infecting species. RVC was significantly more often detected in weaned pigs compared to suckling and fattening pigs, and RVB more often in weaned compared to suckling pigs (Figure 2). RVA was present frequently in all age groups, in weaned and fattening pigs mainly as co-infection with other RV species, and it was therefore not significantly different between age groups.

### 3.2. Longitudinal Study

**Figure 3:**
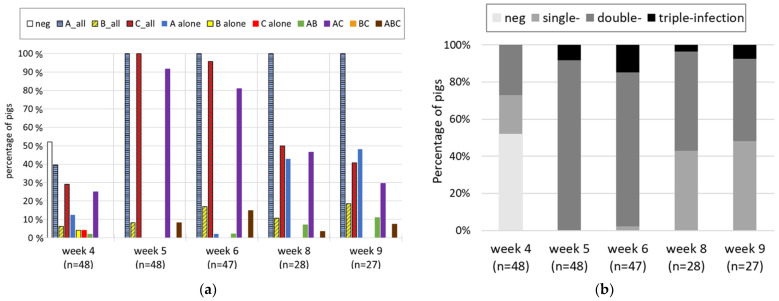
The percentage of pigs from the longitudinal study with (**a**) different types of RV (co-) infections and (**b**) different numbers of (co-)infecting RV species are shown, grouped by the sampling week (=week after birth). The piglets were weaned in week 4. The “_all” indicates that all samples positive for this RV species are included in this group (=overall result), independent of co-infecting species.

In week 4 after birth, when the suckling piglets (*n* = 48) of five litters were weaned, 52% were negative for all tested RV species. The other half were found to be positive mainly for RVA overall (40%), followed by RVC (29%) and only a few for RVB (6%) (Figure 3a). When splitting the overall results into the different types of (co-)infections, the combination of RVA and RVC was most frequent (23%), followed by RVA alone (13%). RVC and RVB single infections were rare, with 2 two animals each (4%); a single animal had an RVA and RVB double infection, and no triple infections were observed. In week 5, all animals were positive for RVA overall and RVC overall, but none of them was a single infection. In 92% of the animals, RVA and RVC double infections were observed, and in 8%, triple infections were observed. In the following weeks, all animals remained positive for RVA overall, but the percentage of RVA and RVC double infections decreased, while the RVA single infections increased. Besides for week 4, no RVC and RVB single infections and never an RVB and RVC double infection were detected throughout the study. In summary, already in week 4, double infections were frequent and became the main type of infection in weeks 5 and 6 but decreased after week 6, when single infections increased again (Figure 3b). In contrast to the cross-sectional study, where triple infections were dominant, they were less frequent in this study. They showed up in week 5 and peaked in week 6 but were never found in more than 15% of the animals.

The five sows and the sow pen floor were sampled when separating the piglets in week 4. The fecal samples of the sows were negative for RVA and RVB, but two animals were weakly positive for RVC. In contrast, the five sow-pen floor samples, which also contained feces from the piglets, were all weakly positive for RVA: two for RVA alone; two for RVA and RVC; and one for RVA, RVB, and RVC. Both weaning pens were weak positive for RVA before the piglets were placed there.

**Figure 4:**
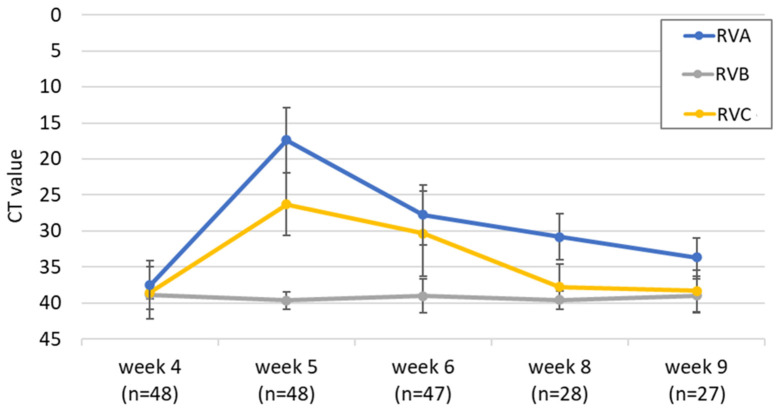
Mean Ct values for RVA, RVB, and RVC and the respective standard deviations of samples from the longitudinal study are shown, grouped by the sampling week (=week after birth). The piglets were weaned in week 4.

Not only did the percentage of RV-positive animals increase dramatically upon weaning, but so did the detected RNA levels (Figure 4). The RVA Ct values decreased (i.e., the viral RNA load increased) in all animals upon weaning and ranged from 15 to 20 in week 5. After this peak, the RNA levels decreased again but remained above the values of week 4. The development of the Ct values of all animals is provided in Supplementary Figure S2. The picture was similar for RVC, where a clear peak was observed in week 5, but the mean Ct value remained below the one for RVA at all time points. In contrast, no peak was visible after weaning for RVB, and the viral RNA load was generally low. However, the number of RVB-positive samples was also considerably lower than for the other two RV species (Figure 3a). When collecting the fecal samples, the presence of diarrhea was also monitored. No animal was diarrheic in week 4. Interestingly, despite the high RVA and RVC RNA load in week 5, only six weaned pigs showed diarrhea at the sampling day: two animals showed severe and four showed mild diarrhea. Mild diarrhea was also observed in week 6 (*n* = 2), week 8 (*n* = 7), and week 9 (*n* = 3).

### 3.3. NGS and Genotyping of RVA Strains

In order to determine the full-genome sequence, RVA-positive fecal samples of the cross-sectional study with Ct values below 33 were subjected to NGS. Of the 29 samples included, 11 did not result in any segment being covered more than 50%. In a further 10 samples, the majority of segments did not fulfill the criteria for genotyping (i.e., sequence of >500 nt and >50% of CDS covered [22]). In one sample, all segments were typable with the CDS being covered between 60.9% and 100% (Supplementary Table S2). In the case of the remaining seven samples, all segments were typable and showed CDS coverages of over 95%. Only the eight samples that fulfilled the genotyping criteria for all segments were included in further analyses. The number of generated total reads ranged between 8.4 and 25.4 Mio for these eight samples; the absolute number of reads matching to the respective strain (summing up the reads matching to each segment) ranged between 13.434 and 2.2 Mio, resulting in 0.08% and 8.5% of relative matching reads, respectively (Supplementary Table S2). While the sample with the lowest relative read number also had the lowest RVA RNA load, there was no general correlation between Ct value, relative read numbers, and CDS coverage observed for the eight samples.

Determination of the full-genome genotypes of the eight strains by the RotaC online typing tool revealed a common R1-C1-M1-I5-A8-N1-H1 backbone for VP1, VP2, VP3, VP6, NSP1, NSP2, and NSP5, respectively, for all samples (Table 1). For NSP3, most sequences were of the T7 genotype, but in two cases, T1 was also present. Similarly, for NSP4, the E1 genotype was prevailing, but, in two samples, E9 was found. These backbone genotypes are the same as observed in the Belgian reference strains (Table 1). For VP7, five different genotypes were present: the G9 genotype was most frequently observed (*n* = 5), followed by G5 (*n* = 4), G4 (*n* = 2), G3, and G11 (one sample each). With exception of G11, these genotypes are also present in the Belgian reference genomes. G11 is present in the porcine reference isolate YM. For VP4, four different genotypes were found: P[13] was most frequent (*n* = 5), followed by P[6] (*n* = 4), P[7], and P[32] (*n* = 2 for both). While P[13], P[6], and P[7] are also present in well-known porcine reference genomes, P[32] has so far only been described in pigs from Ireland, UK, Denmark, and Germany [36,37,38,39,40,41,42,43,44,45,46].

Interestingly, we found, in four cases, an indication of the presence of multiple RVA strains, while the other four seemed to be single infections with a single strain. In the case of VP4, up to four different genotypes were observed in the same sample for VP7 up to three. In these cases, the backbone segments also showed multiple sequences but usually of the same genotype. There were, for example, four different sequences of the genotype T7 (NSP3) present in S18-1093.

**Figure 5:**
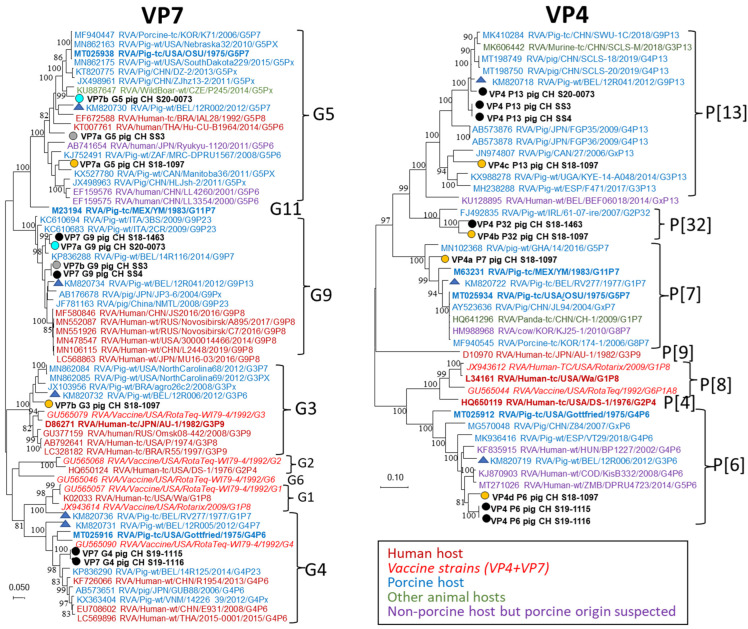
Maximum likelihood phylogenetic trees of the CDS of VP7 and VP4 sequences. RVA strains from this study are highlighted with circles, and names are written in bold black letters. Circle fill-colors gray, blue, and yellow indicate the common origin of co-infecting sequences. Letters after the protein ID of the Swiss strains (e.g., VP7a and VP7b) indicate multiple sequences for the CDS of this segment. Names of human reference strains are written in dark red, vaccine strains in light red and italic, porcine reference strains in blue, and strains from other animals in green. Viral sequences gained from non-porcine hosts but suspected to originate from pigs through interspecies transmission or following reassortment are marked in purple. The porcine lab strains included in Table 1 are highlighted in bold, and the Belgian reference strains from Table 1 are highlighted with blue triangles.

**Figure 6:**
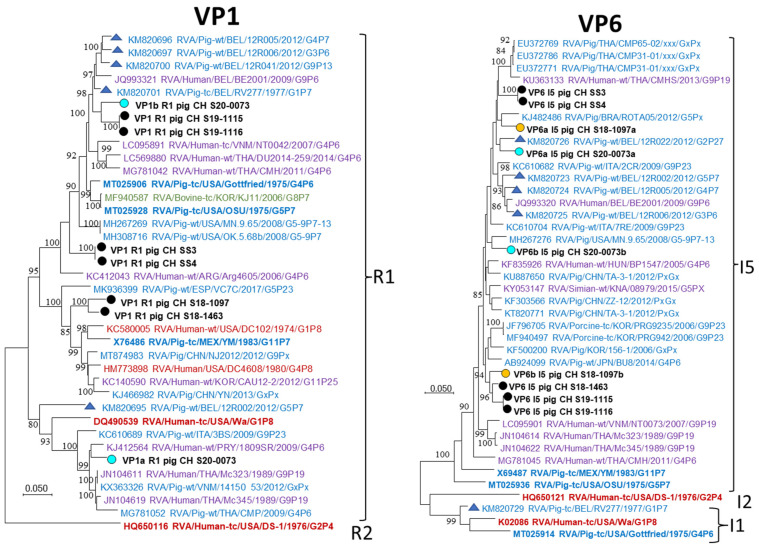
Maximum likelihood phylogenetic trees of the CDS of VP1 and VP6 sequences. RVA strains from this study are highlighted with circles, and names are written in bold black letters. Circle fill-colors blue and yellow indicate the common origin of co-infecting sequences. Letters after the protein ID of the Swiss strains (e.g., VP1a and VP1b) indicate multiple sequences for the CDS of this segment. The reference strains included in Table 1 are highlighted in bold, and Belgian references with triangles. A legend of the color codes of the strain names is provided in Figure 5.

**Figure 7:**
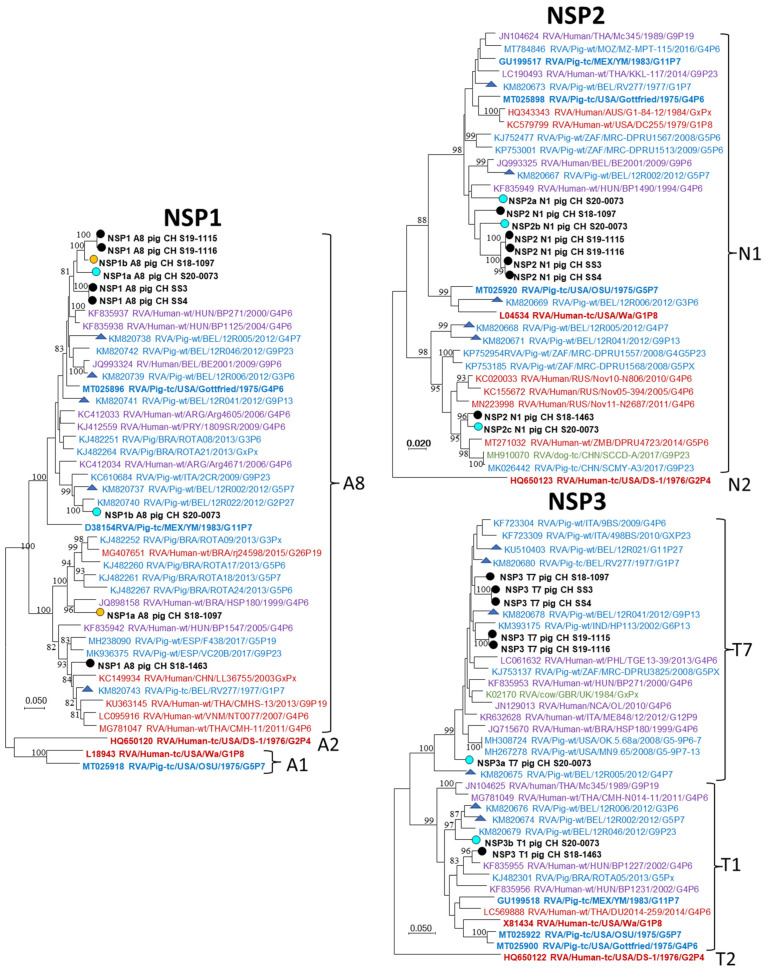
Maximum likelihood phylogenetic trees of the CDS of NSP1, NSP2 and, NSP3 sequences. RVA strains from this study are highlighted with circles, and names are written in bold black letters. Circle fill-colors blue and yellow indicate the common origin of co-infecting sequences. Letters after the protein ID of the Swiss strains (e.g., NSP1a and NSP1b) indicate multiple sequences for the CDS of this segment. The reference strains included in Table 1 are highlighted in bold and Belgian references with triangles. A legend of the color codes of the strain names is provided in Figure 5.

All sequences of the seven samples where every CDS was covered >95% were used for phylogenetic analyses. Figure 5, Figure 6 and Figure 7 show the phylogenetic trees of the segments where suspected co-infection with several RVA strains was observed (VP7, VP4, VP1, VP6, NSP1, NSP2, and NSP3), while the trees of segments where only single sequences were found (VP2 and VP3; NSP4 and NSP5) are presented as Supplementary Figure S3. For VP7 and VP4, the Swiss strains were more closely related to porcine than human reference sequences, including the Belgian references, or strains that were isolated from humans but are thought to be of porcine origin (Figure 5). Two pairs of samples, S19-1115 and S19-1116, and SS3 and SS4 originated from the same pig herds and showed identical or near-identical sequences. The remaining Swiss sequences did not form distinct clusters. For P[32], only a single fully sequenced reference genome was available.

For VP1 and VP6 only single genotypes, R1 and I5, respectively, were found in the analyzed Swiss pigs but in both cases multiple sequences were detected in the same samples (Table 1, Figure 6). The suspected co-infecting sequences showed a comparably high genetic distance. All sequences were most closely related to references of porcine origin but did not form distinct clusters. For VP2 and VP3 only single sequences of the C1 and M1 genotypes, respectively, were detected (Supplementary Figure S3). In contrast to VP1 and VP6, the Swiss strains were closely related, with exception of single “outliers”: S18-1097 within C1 and S19-1115/-1116 within M1.

In three of the five segments coding for non-structural proteins (NSP) multiple simultaneous sequences were observed. In case of NSP1 only A8 sequences were found and the two sample pairs S19-1115/-1116 and SS3/-4 were closely related. Together with one each of the sequences from S18-1097 and S20-0073 they seemed to form a distinct cluster within A8. S18-1463 and the second sequences of S18-1097 and S20-0073 were more distantly related. A similar situation is observed for NSP2 and NSP3 where most Swiss sequences were closely related except for S18-1463 and single S20-0073 sequences. In contrast, no clustering could be observed for NSP4 and NSP5 sequences. Similar to the segments coding for structural proteins, the non-structural protein coding sequences were generally more closely related to references of porcine than human origin.

## 4. Discussion

### 4.1. Cross-Sectional Study

The fecal samples of 89% of the 95 pigs included in this study were positive for at least one RV species. This prevalence is higher than the 3–63.7% reported by Vlasova et al. for RVA globally [2]. However, this number may not be representative for the entire Swiss pig population, as the samples were provided by the Institute of Pathology and, hence, may originate mainly from herds with health problems. Interestingly, over two-thirds of the samples were positive for several RV species. The most frequent type of co-infections were triple infections with RVA, RVB, and RVC and double infections with RVA and RVC, while RVB and RVC co-infections were rare. This finding is comparable to other studies, where triple infections were reported most frequently (12–21.1%), followed by AC (6.7–24%), BC (3–8%), and AB (5–16%) double infections [14,17,42,43]. As also reported previously, single infections were mainly caused by RVA, while RVC and, particularly, RVB were rarely observed alone [15]. Overall, we found a clear correlation between weaned pigs, triple infections, and signs of disease, represented by diarrhea and wasting. As a third measurement of clinical impact, we used the VCR. However, while the percentage of animals with severe villi shortening increased with the number of co-infecting RV species, the difference was not statistically significant, and we saw moderate shortening of the villi also in surprisingly many RV negative animals. The reason for the better correlation of multiple RV infections with diarrhea and wasting may be due to the comparably complex measurement of the VCR, which ideally is performed on several sections of the small intestines, while we had only a single section available. It also requires perfect condition and visualization of the enteral morphology. In addition, other pathogens, such as *Cystoisospora suis*, may lead to shortened villi and may have influenced the result [40]. While we can exclude TGEV and PEDV, the availability of data concerning the presence of other pathogens was inconsistent between samples and was therefore not included. We can also not fully exclude the presence of other RV species. We did not detect RVH reads in the NGS data, but they could be present below the detection limit or in the non-sequenced samples. Nevertheless, our data allowed the hypothesis that suckling piglets are mainly infected by a single RV species, most commonly RVA, but that weaning may trigger infection with (or the shedding of) additional RV species, and this may be paralleled with health impairment.

### 4.2. Longitudinal Study

To gain further data regarding the dynamic of RV species, we retrospectively analyzed samples from a group of piglets that were followed through the weaning phase until the start of the fattening period for another study. In contrast to the cross-sectional study, where most samples originated from farms with overt health problems, this farm had no severe health issues, and this hampers comparison somewhat. However, it still provides valuable information on the dynamic of RV species and temporal occurrence of (co-)infections. In contrast to the cross-sectional study, a relatively high number of piglets with double infections of RVA and C was observed already in week 4, just prior to weaning. Impressively, all animals were shedding RVA and C just one week later. In week 5 and the following weeks, triple infections were also observed but were not as dominant as in the cross-sectional study. The reason for the early presence of RVC is likely due to the viral shedding of at least two out of five sows. Real-time RT-PCR revealed a dramatic peak of shed RVA and C in all animals in the first week after weaning. While the weaning pens were already weak positive for RVA when the piglets were placed there, it is unclear if the virus was infectious since Ct values were very high. It has been shown before that lactogenic IgA and IgG can protect piglets from RV infection [2,41]. After weaning, the piglets are deprived of this protection, thus allowing pre-existing RV infections to propagate and facilitating infection with new RV species. Infections with mixed RV species were previously shown to be associated with diarrhea [14,16,42] and seem to intensify the severity of diarrhea in piglets [14,18]. The results of our cross-sectional study confirm these findings. However, despite of the massively increased shedding of RVA and C in week 5, and in contrast to the cross-sectional study, only a few pigs showed symptoms of diarrhea in the longitudinal study. Since diarrhea in piglets is considered a multifactorial disease, this might be due to favorable general conditions such as the absence of other pathogens in this herd. However, we have no conclusive information on the range of pathogens present on this farm. Furthermore, the five sows were experienced multiparous animals, which are expected to confer a high concentration of anti-RV maternal antibodies to their piglets. As reported by Katsuda et al., piglets from gilts (primiparous sows) had a significantly higher prevalence of RV, while high antibody titers reduced the risk of early onset of RV diarrhea [42]. Another explanation for the comparably low rate of diarrhea could be the fact that the weaned pigs in the longitudinal study showed mostly double infections, whereas, in the cross-sectional study, we found triple infections significantly associated with diarrhea. However, longitudinal data of more farms, also including analysis of additional pathogens and histological findings, are necessary to draw final conclusions.

### 4.3. NGS and Genotyping of RVA Strains

We used a non-specific NGS approach, followed by de novo assembly, to determine the full-genome sequences of RVA strains from the cross-sectional study. Except for VP7 and VP4, the genotypes of the eight fully typed samples were mostly identical and followed the pattern I5-R1-C1-M1-A8-N1-T7-E1-H1, which is frequently found in pigs globally [13,19,42,47]. In two samples, a combination of T1 and E9 instead of T7 and E1 was observed. While T1 is also commonly described in pigs worldwide, E9 is rarer and has, according to the NCBI rotavirus database, only been shown in pigs from Ireland, Canada, Belgium, and Spain [37,38]. For VP7 and VP4, the diversity was higher, with five different VP7 genotypes (G9 > G5 > G5 > G3/G11) and four different VP4 genotypes (P[13] > P[6] > P[7] > P[32]). A recent study in Germany has resulted in a similar genotype diversity and frequency [37]. However, we did not observe the VP4 genotype P[23], which was most frequently detected in Germany, but our samples are far from being representative for the full RV diversity present in Swiss pigs. Interesting is also the detection of P[32] in Swiss pigs which is frequent in Germany but, else, has only been reported from Ireland, the UK, and Denmark [36,37,38,39], and, up to now, only the Irish strain was fully sequenced. Due to the low number of samples, it is difficult to conclude on the presence of Swiss subcluster within RV genotypes. However, except for NSP1, NSP2, VP2, and VP3, where most Swiss sequences are closely related, the phylogenetic trees provide no evidence for Swiss-specific subclusters of RVA, as previously observed for other porcine viruses such as hepatitis E virus (HEV) or atypical porcine pestivirus (APPV) [43,44]. In those cases, the high restrictions for the import of living pigs into Switzerland are thought to have favored virus evolution independent from neighboring countries. Due to the high tenacity of RVA, indirect transmission may be more frequent than in case of HEV or APPV and could explain cross-border exchange of viruses in the absence of pig movement.

Interestingly, 50% of our fully sequenced samples showed the presence of several RV strains, making it impossible to determine the exact G–P combinations. While we cannot fully exclude cross-contamination during the postmortem analysis, the sequencing approach we used is unlikely to allow for minor contaminations to result in typable sequences. Infections with multiple RV strains have been shown before, e.g., in human stool samples from Africa and Taiwan, and are hypothesized to contribute to lowered vaccine efficiency [19,45]. Nyaga et al. also provided evidence for simultaneous infection with human and presumed animals RV strains that may give rise to new reassortants [19]. Hull et al. speculated that the live RVA vaccines widely used in the USA may promote RVA diversity and give rise to multiple infections [13]. However, since no RVA vaccines are available in Switzerland, we can exclude this explanation. To our knowledge, multiple RV strains in fully sequenced porcine RVA have so far only been shown in single cases by Hull et al. in the USA and Phan in Vietnam [13,45]. Our results indicate that they may occur more frequently than thought and may encompass up to four different RVA strains. Interestingly, all pigs with multiple RVA infections were also infected with RVC and RVB, were five or six weeks old, and suffered from diarrhea and wasting. Hence, one might hypothesize that the weaning process may not only facilitate infection of multiple RV species but also of multiple strains of the same species. Age-related changes in the histo-blood group antigens (HBGAs), which serve as virus (co-)receptors for some RVA genotypes, were shown to influence susceptibility to RVA in children [46]. Not much is known about the relevance of HBGA in porcine RV infection. Experiments using the porcine G9P[13] genotype point toward contrasting roles of HBGA and sialic acids for porcine and human RV [47]. Studies on age- and/or weaning-induced changes of the porcine intestinal glycans and the receptor usage of different porcine RV genotypes might help to better understand the susceptibility of weaned piglets for multiple RV infections.

## 5. Conclusions

Clearly, further studies including samples representative for the entire Swiss pig population, as well as longitudinal studies including more herds, e.g., of different sizes and production types, with and without RV-associated health problems, are necessary to draw final conclusions on the RV infection dynamics. However, the cross-sectional and longitudinal studies presented here provide some valuable first information on the occurrence and genetic diversity of porcine RVA, RVB, and RVC in Switzerland. The results of both studies indicate weaning to be the main trigger for increased shedding of RV, both qualitatively and quantitatively. In addition, weaning also seems to favor infection with multiple RV species, and this was associated with diarrhea and wasting. While the diversity of RVA genotypes was similar to that seen in pigs in other European countries, co-infection with up to four RVA strains simultaneously raises concerns about the generation of new, possibly zoonotic, reassortants. Hence, identifying factors that influence susceptibility for RV infection upon weaning might lower not only losses due to RV diarrhea and the risk for the generation of novel reassortants but also unnecessary antibiotic treatment in the case of diarrhea problems.

## Figures and Tables

**Table 1:** RVA genotypes of strains from this study (bold) and reference strains. Wa-like genotypes are colored green, SD-1-like red, Au-1-like orange, typical porcine genotypes blue, and mixed or unclear genotypes are uncolored.

Strain, Host	VP7	VP4	VP6	VP1	VP2	VP3	NSP1	NSP2	NSP3	NSP4	NSP5	Reference
SS3, pig *	G9	P[13]	I5	R1	C1	M1	A8	N1	T7	E1	H1	
	G5											
SS4, pig *	G9	P[13]	I5	R1	C1	M1	A8	N1	T7	E1	H1	
S19-1115, pig *	G4	P[6]	I5	R1	C1	M1	A8	N1	T7	E1	H1	
S19-1116, pig *	G4	P[6]	I5	R1	C1	M1	A8	N1	T7	E1	H1	
S18-1463, pig *	G9	P[32]	I5	R1	C1	M1	A8	N1	T1	E9	H1	
S18-1097, pig *	G5	P[13]	2 × I5	R1	C1	M1	2 × A8	N1	T7	E1	H1	
		P[6]										
	G3	P[7]										
		P[32]										
S20-0073, pig *	G5	P[13]	2 × I5	2 × R1	C1	M1	2 × A8	3 × N1	T1	E9	H1	
	G9								T7			
**S18-1093, pig**	G5	P[13]	3 × I5	2 × R1	2 × C1	M1	3 × A8	N1	4 × T7	2 × E1	H1	
	G11	P[6]										
	G9	P[7]										
12R002, pig	G5	P[7]	I5	R1	C1	M1	A8	N1	T1	E1	H1	[35]
12R005, pig	G4	P[7]	I5	R1	C1	M1	A8	N1	T7	E1	H1	
12R006, pig	G3	P[6]	I5	R1	C1	M1	A8	N1	T1	E1	H1	
12R041, pig	G9	P[13]	I5	R1	C1	M1	A8	N1	T7	E1	H1	
RV277, pig	G1	P[7]	I1	R1	C1	M1	A8	N1	T7	E1	H1	
Gottfried, pig	G4	P[6]	I1	R1	C1	M1	A8	N1	T1	E1	H1	[22]
OSU, pig	G5	P[7]	I5	R1	C1	M1	A1	N1	T1	E1	H1	
YM, pig	G11	P[7]	I5	R1	C1	M1	A8	N1	T1	E1	H1	
Wa, human	G1	P[1]	I1	R1	C1	M1	A1	N1	T1	E1	H1	
DS-1, human	G2	P[2]	I2	R2	C2	M2	A2	N2	T2	E2	H2	
Au-1, human	G3	P[3]	I3	R3	C3	M3	A3	N3	T3	E3	H3	

* Sequences presented in the phylogenetic trees.

## Data Availability

The sequences generated in this study are available in NCBI GenBank under the accession numbers OM982707–OM982822; details are provided in Supplementary Table S1. NGS raw data are deposited in NCBI Sequence Read Archive (SRA) as BioSamples SAMN26634154–SAMN26634161.

## Supplementary Materials

The following are available online at https://www.mdpi.com/article/10.3390/v14051117/s1. Figure S1: Depiction of the histological measurement of the VCR. Figure S2: Chart of the development of the Ct values for RVA, RVB, and RVC of all animals of the longitudinal study. Figure S3: Phylogenetic trees of VP2, VP3, NSP4, and NSP5. Table S1: Accession numbers of all sequences included in Table 1. Table S2: Detailed description of the samples included in Table 1.
