# Supplementary material for: Frequent Occurrence of Simultaneous Infection with Multiple Rotaviruses in Swiss Pigs
Source: Viruses. 2022 May 23;14(5):1117. doi: 10.3390/v14051117 (PMC9147839; doi:10.3390/v14051117)
Supplement: Supplementary file 1 [file viruses-14-01117-s001.zip › viruses-1700268-supplementary.pdf]

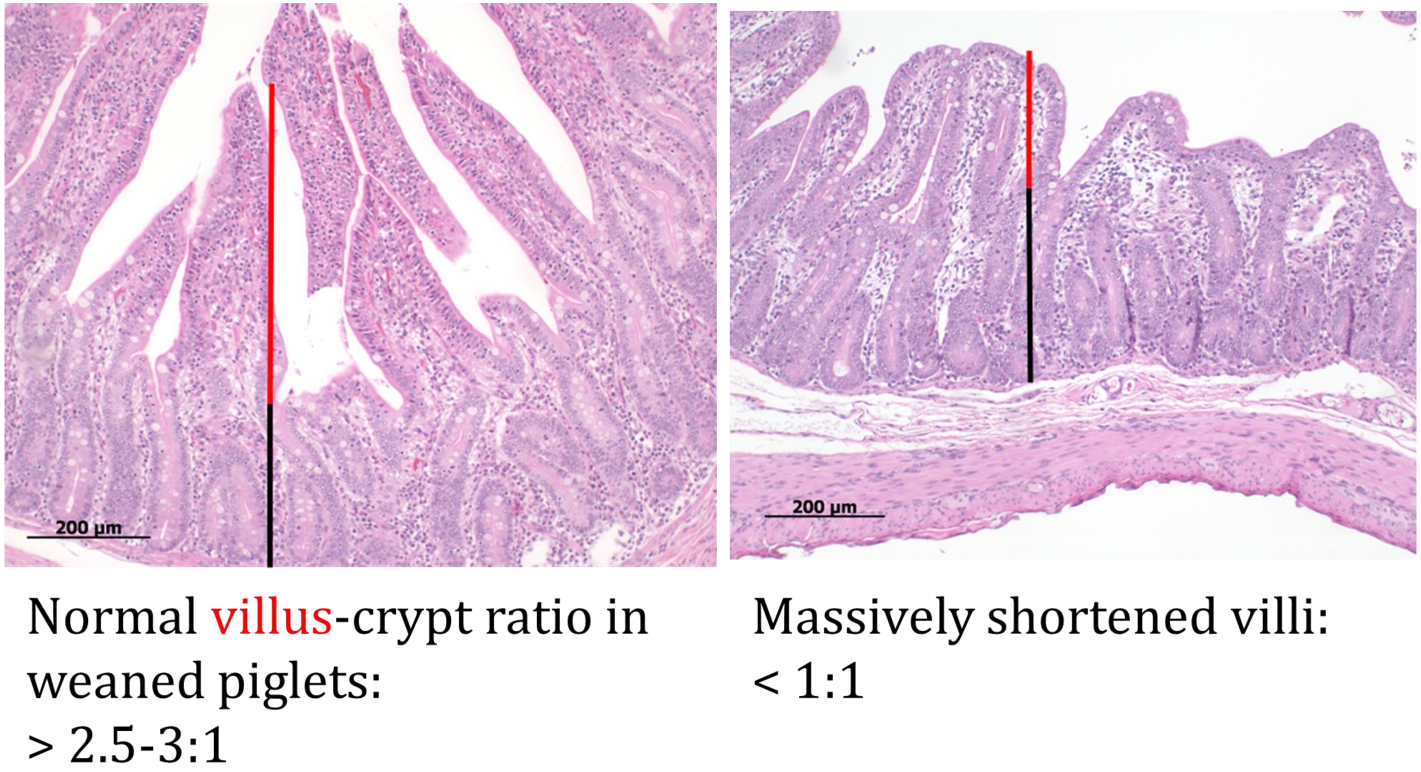

Figure S1. HE-stained section of the jejunal mucosa showing the measurement of the villus-crypt ratio (VCR). The left picture shows the normal VCR, the picture on the right shows a severely reduced VCR in a pig infected with RV.

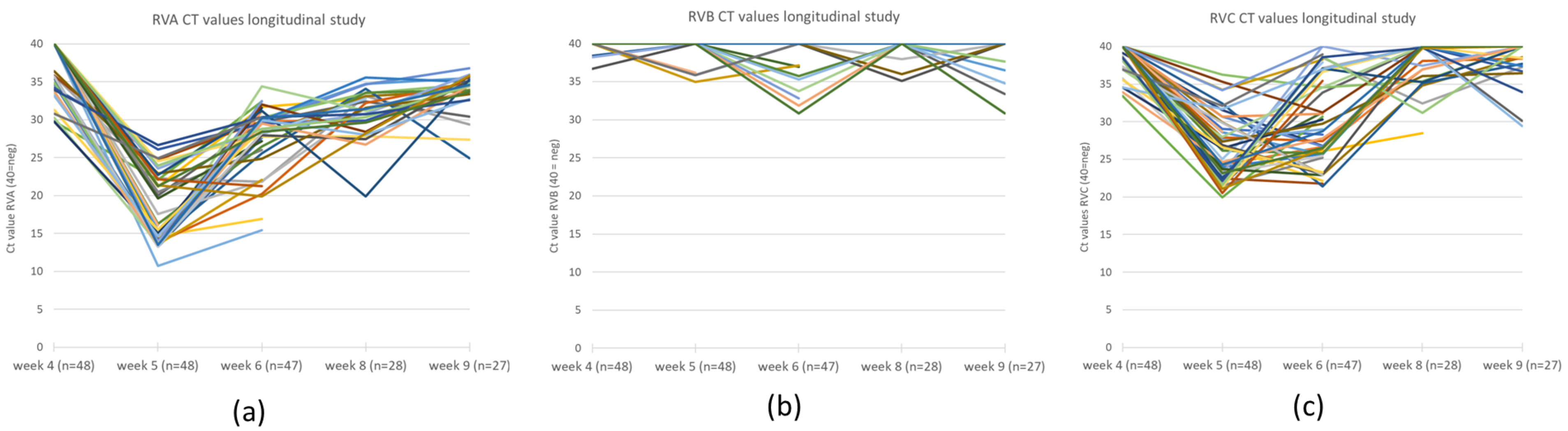

Figure S2. Ct values of individual animals included in the longitudinal study for (**a**) RVA, (**b**) RVB and (**c**) RVC. The maximum cycle number was 40.

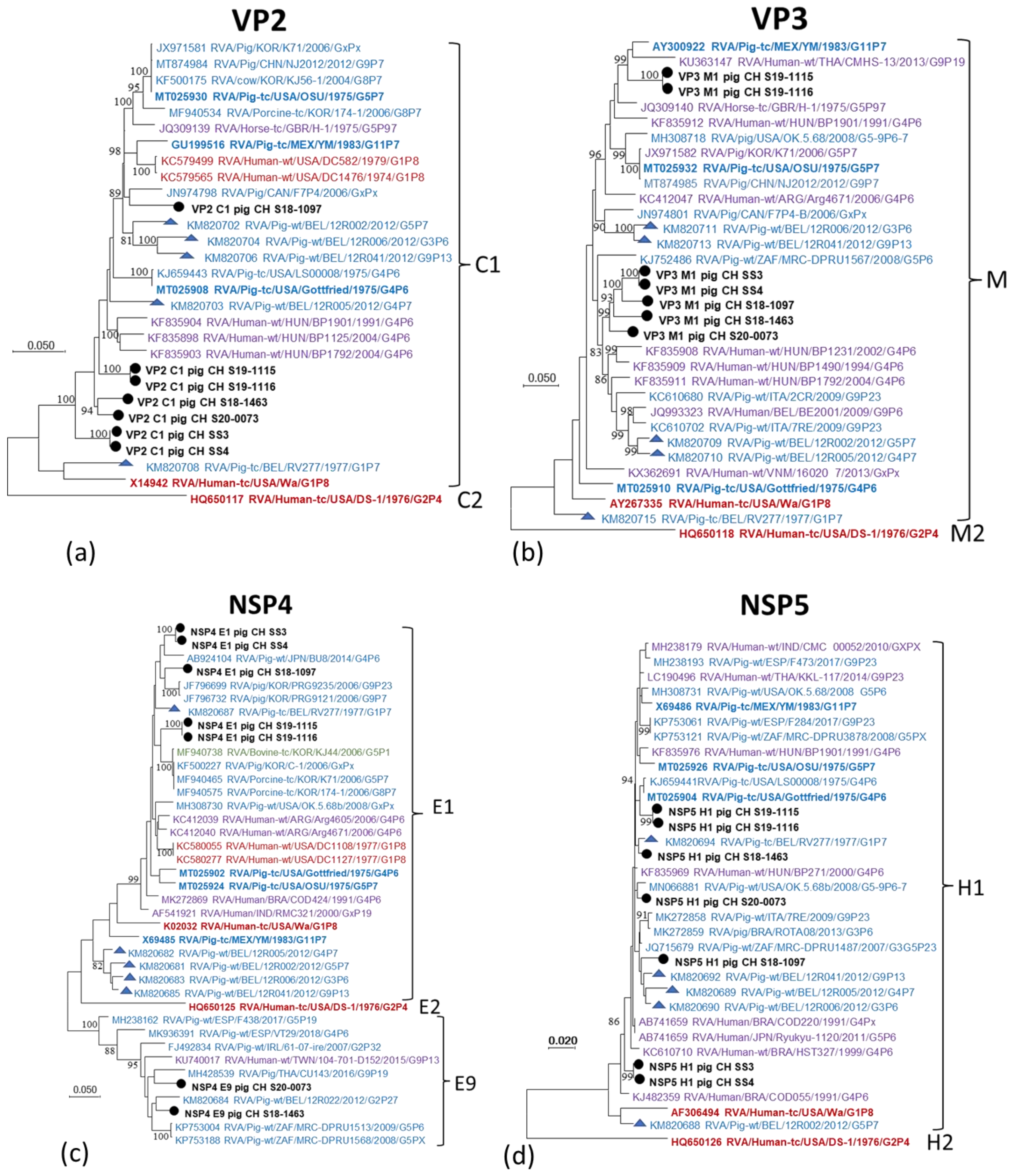

Figure S3. Maximum likelihood phylogenetic trees of the CDS of (**a**) VP2, (**b**) VP3, (**c**) NSP4, (**d**) NSP5 sequences. RVA strains from this study are high-lighted with circles and names are written in bold black letters. Names of human reference strains are written in dark red, porcine reference strains in blue and other animals in green. Viral sequences gained from non-porcine hosts but suspected to originate from pigs through interspecies transmission or following reassortment are marked in purple. The porcine lab strains included in Table 1 are highlighted in bold and the Belgian reference strains from Table 1 are highlighted with blue triangles. A legend for the color codes of the strain names is provided in Figure 5.
